# Homeostasis and failure of mitochondria on the single-cell level

**DOI:** 10.4103/NRR.NRR-D-25-00708

**Published:** 2025-10-30

**Authors:** Kristina Friedland, Kristina Endres

**Affiliations:** Institute for Pharmaceutical and Biomedical Research, Johannes Gutenberg-University Mainz, Mainz, Germany; University of Applied Sciences Kaiserslautern, Campus Zweibrücken, Zweibrücken, Germany; Department of Psychiatry and Psychotherapy, University Medical Center of the Johannes Gutenberg-University, Mainz, Germany

Mitochondria are the central organelles that allow eukaryotic cells to efficiently convert nutrients into energy for cellular functions such as anabolic reactions, movement, and regulation. A reduction in the number of mitochondria or the occurrence of dysfunctional mitochondria leads to serious diseases such as the Leigh syndrome. However, such changes have also been connected to Alzheimer’s disease (AD) and many more diseases of different organ systems and occur during the aging process. Mitochondria are, therefore, the linchpin for the homeostasis of individual cells but also lay the foundation for a healthy, functional organism as a whole. Tissues and organs that consume a lot of energy show a loss of function early on if the mitochondria fail. This includes the brain, but also other organs such as the heart or the intestines, which have to provide high performance or are subject to permanent renewal. The importance of mitochondria for the integrity of neurons of the brain may become clear when looking at the almost altruistic behavior of microglia: a transfer of mitochondria into neurons was observed, for example, when neurons were exposed to α-synuclein or tau, hallmarks of Parkinson’s disease or AD (Scheiblich et al., 2024). By establishing tunneling nanotubes, healthy microglia donated the organelles to affected neurons and rescued them by reducing reactive oxidative species. Mitochondria comprise highly dynamic organelles that adjust their shape, location, and number by processes such as fusion and fission. Thus, local events such as noxae might imprint fast on them to serve actual cellular demands. In this perspective, we will focus on recent research regarding mitochondrial failure in AD and on how novel techniques that allow spatial resolution in analyzing these organelles contribute to widening the knowledge in this regard.

**Mitochondrial dysfunction in Alzheimer’s disease:** AD is the most prevalent form of dementia in the elderly, accounting for approximately 70% of all dementia cases. It imposes a considerable burden of suffering not only on patients themselves but also on their caregivers, who are often close relatives. Despite decades of research, the pathogenesis of AD remains largely elusive (Scheltens et al., 2021). The majority of cases (up to 99%) are classified as sporadic, with no clearly defined genetic cause. However, increasing evidence suggests that mitochondrial dysfunction may be a hallmark of the disease. One of the earliest pathological changes observed in AD brains is impaired glucose metabolism. As neurons depend heavily on oxidative phosphorylation of glucose to generate adenosine triphosphate, this hypometabolism indicates a deficit in mitochondrial function. In contrast, astrocytes and oligodendrocytes predominantly meet their energy demands through aerobic glycolysis. Microglia primarily rely on oxidative phosphorylation under physiological conditions, but neuroinflammation can reprogram them toward a glycolysis-dominant metabolic phenotype (Cunnane et al., 2020).

Previous studies have demonstrated reduced mitochondrial function in neurons and microglia in AD (Jörg et al., 2021; Li et al., 2022). This is evidenced by decreased expression of subunits of respiratory chain complexes, particularly complexes I, III, and IV, which is associated with diminished mitochondrial respiration (Askenazi et al., 2023). Additionally, altered mitochondrial morphology—characterized by fragmentation, disrupted cristae structure, impaired fusion, and increased fission—as well as defective mitophagy, have been observed in various models, including neuronal and microglial cultures, human induced pluripotent stem cell-derived neurons from AD patients, animal models, and post-mortem human brain tissue.

The precise cause of mitochondrial dysfunction in AD is still under debate. Some researchers propose that age-related mitochondrial decline leads to elevated production of reactive oxygen species, which in turn activates the amyloidogenic pathway. Within this pathway, a type I transmembrane protein, the Amyloid precursor protein, is proteolytically processed by two proteases close to and within the membrane stalk. This results in formation of toxic amyloid-beta (Aβ) monomers that subsequently can form oligomers and finally deposit as plaques. Besides this rather indirect effect of reactive oxygen species on Aβ synthesis, others suggest that Aβ itself directly impairs mitochondrial function. These findings point to a vicious cycle in which mitochondrial dysfunction and Aβ accumulation exacerbate each other, ultimately leading to neuronal damage. In addition to Aβ, hyperphosphorylated tau has also been shown to impair mitochondrial function. Hyperphosphorylated tau is – besides the aggregating Aβ peptides – a fundamental hallmark of AD but filamentous inclusions of this protein also occur in other neurological diseases such as Frontotemporal dementia or Pick’s disease, summarized as tauopathies. In neuronal cell lines, induced pluripotent stem cell-derived neurons, and astrocytes, tau pathology has been associated with reduced complex I and ATPase activity, altered mitochondrial morphology and ultrastructure, decreased fusion, and increased fission (Parra Bravo et al., 2024).

However, most of these investigations have been conducted using bulk analyses, such as plate-reader-based assessments of mitochondrial membrane potential or oxygen consumption. While useful, such approaches average out individual cellular differences and obscure subcellular heterogeneity. Imaging techniques like confocal microscopy, electron microscopy, and high-resolution microscopy allow for the visualization of mitochondrial ultrastructure at the single-organelle level but do not capture functional dynamics across populations of cells.

**Investigation of single-cell based mitochondrial function:** Recent advances in single-cell technologies have opened new avenues for investigating mitochondrial function at unprecedented resolution. Single-cell RNA sequencing and spatial transcriptomics enable the characterization of mitochondrial gene expression at the individual cell level. However, even these techniques can overlook mitochondrial heterogeneity within a single cell. For instance, mitochondria within one cell may vary significantly in morphology, motility, mitochondrial DNA content, and protein expression. To overcome this limitation, nanobiopsy has emerged as a minimally invasive, high-precision technique that allows molecular interrogation of individual subcellular compartments without destroying the cell (Bury et al., 2024). This method has enabled spatially resolved analysis of mitochondrial DNA heteroplasmy in situ. For example, a proof-of-concept study combined nanobiopsy with next-generation sequencing to analyze subcellular mitochondrial DNA heteroplasmy in human tissues (Bury et al., 2024). Nevertheless, these techniques need highly experienced personnel and a self-established, complex equipment. Building on the principles of cell-specific extraction procedures, the commercially available Single Cellome™ System SS2000 enables the extraction of intact, functional mitochondrial material from single cells, coupled with real-time visualization using confocal microscopy. This system exemplifies the practical translation of small-scale biopsies into a user-friendly platform for single-cell mitochondrial analysis. Complementing these experimental techniques are computational tools developed to dissect mitochondrial heterogeneity from single-cell transcriptomic data. Platforms such as MitoTrace, MQuad, and MAESTER facilitate the identification and analysis of mitochondrial variants in single-cell RNA sequencing datasets.

In conclusion, single-cell-based methodologies, including transcriptomics, nanobiopsy, and computational approaches, provide critical insights into mitochondrial heterogeneity and function. These techniques transcend the limitations of traditional bulk analyses and have the potential to revolutionize our understanding of mitochondrial biology in AD. Moreover, such advancements may pave the way for novel diagnostic and therapeutic strategies tailored to mitochondrial alterations in individual cells. Techniques that combine the visualization of mitochondria alongside Aβ oligomers or Tau fibrils in single cells, followed by single-cell extraction and subsequent sequencing, are of particular interest. These methods enable the direct localization of Aβ oligomers and Tau tangles at the organelle level—specifically close to or within mitochondria in individual cells. This integrated approach will allow the assessment of how Aβ oligomers and Tau tangles affect mitochondrial morphology or fission and fusion in distinct cell populations and will provide deeper mechanistic insights into their detrimental impact on mitochondrial function.

**Figure 1:**
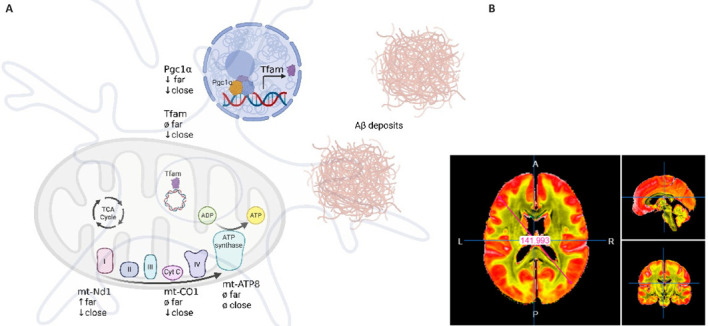
Spatial analysis of mitochondrial function and distribution. (A) Extraction of mitochondria from microglia in close and farther distance from Aβ peptide deposits indicated a spatial reactivity pattern that affected components of OXPHOS but also of mitochondrial biogenesis. Created with BioRender.com (WX28PFEX4L). (B) The brain image was derived from ID840206 (https://identifiers.org/neurovault.collection:16418) and consists of a probabilistic map of mitochondria derived from frozen human brain specimen. The developers of the map reported, e.g., a 50% higher content of mitochondria in grey matter as compared to white matter indicated here by red *versus* yellow labelling (Mosharov et al., 2025). Aβ: Amyloid-beta; CytC: cytochrome C; mt-CO1: cytochrome c oxidase I; mt-ND1: NADH dehydrogenase 1; Pgc1α: peroxisome proliferator-activated receptor γ coactivator 1α; TCA cycle: tricarbonic acid cycle; Tfam: mitochondrial transcription factor A.

**Spatial dissection of mitochondrial dysfunction in microglia corresponding to proximity to amyloid deposits:** According to the highly dynamic behavior of mitochondria and the fact that they seemingly contribute to AD pathology, gaining insight into local events is mandatory to understand the response of single cell types to the molecular noxae that are pivotal to the disease. In a recent study, we therefore tried to answer the question whether vicinity to Aβ-containing extracellular deposits affects microglia. For this, we extracted nuclei and mitochondria from SIM-A9 microglia close to such deposits by using an SS2000 (combined confocal microscope with aspiration unit) and compared them to cells that were further away (Subirana Slotos et al., 2025). Indeed, we found deviating patterns of gene expression when investigating both nuclear genes and mitochondrial genes that together regulate mitochondrial function (**Figure 1A**). The peroxisome proliferator-activated receptor γ coactivator 1α, the master regulator of mitochondrial biogenesis, for example, was down-regulated, independently of distance to the deposit. On the contrary, the mitochondrial transcription factor A that regulates mitochondrial transcription and participates in mitochondrial genome replication, was only down-regulated in such cells that were found close to the toxic compound. Mitochondrial DNA was, to give another example, only increased in samples from cells close to the deposits. This indicates that not only length of exposure but also spatial resolution can lead to defined reactivity of cells and this reflects on the mitochondrial behavior, coping with the demand of each single cell. This technique would be even more valuable when considering co-cultures of different cell types in the central nervous system and the ability to extract and compare, e.g., mitochondria from a neuron in contact with a plaque-like deposit but supported by astrocytes or facing the toxic component without support.

To add a further layer of complexity, just recently an atlas of mitochondrial phenotypes throughout the human brain has been published (MitoBrainMap; Mosharov et al., 2025). The predicted map was derived from measures based on a single slice and was interpolated to the whole brain by using a machine learning algorithm. This provides a basis for further exploring the dynamics and contributions of mitochondria in diseases such as AD (**Figure 1B** provides an exemplary map). By applying a physical voxelization approach, the developers of this map assigned to cortical and subcortical voxels of frozen human brain parameters that profiled mitochondrial types, based on e.g., OXPHOS enzyme activity (using three markers: complex I (NADH-ubiquinone oxidoreductase), complex II (succinate dehydrogenase) and complex IV (cytochrome c oxidase) but also mitochondrial DNA density. Tissue respiratory capacity and mitochondrial respiration capacity correlated, for instance, with the estimated phylogenetic age of brain regions, reflecting the increased demand of energy in a later evolutionary stage. Selected voxels were furthermore used for single-nuclei RNA sequencing and revealed that mitochondrial phenotypes from different cell types in one brain region were more similar than that of the same cell types in different regions. This again underlines that spatial resolution is mandatory for unraveling mitochondrial (dys-)function in a non-homogeneous environment such as tissue. In the future, preparing such a map based on different stages of neurodegenerative diseases, together with single-cell techniques, will provide an immense insight into mitochondrial dysfunction as being central to these diseases.
